# Imaging African trypanosomes

**DOI:** 10.1111/pim.12046

**Published:** 2013-09-04

**Authors:** L MacLean, E Myburgh, J Rodgers, H P Price

**Affiliations:** 1 Centre for Immunology and Infection, Department of Biology/Hull York Medical School, University of YorkHeslington, York, UK; 2 Wellcome Trust Centre for Molecular Parasitology, Institute of Infection, Immunity and Inflammation, College of Medical, Veterinary & Life Sciences, University of GlasgowGlasgow, UK; 3 Institute of Infection, Immunity and Inflammation, College of Medical, Veterinary & Life Sciences, University of GlasgowGlasgow, UK

**Keywords:** animal model, electron microscopy, *in vivo* imaging, RNA interference, tools and techniques, Trypanosoma spp

## Abstract

*Trypanosoma brucei* are extracellular kinetoplastid parasites transmitted by the blood-sucking tsetse fly. They are responsible for the fatal disease human African trypanosomiasis (HAT), also known as sleeping sickness. In late-stage infection, trypanosomes cross the blood–brain barrier (BBB) and invade the central nervous system (CNS) invariably leading to coma and death if untreated. There is no available vaccine and current late-stage HAT chemotherapy consists of either melarsoprol, which is highly toxic causing up to 8% of deaths, or nifurtimox–eflornithine combination therapy (NECT), which is costly and difficult to administer. There is therefore an urgent need to identify new late-stage HAT drug candidates. Here, we review how current imaging tools, ranging from fluorescent confocal microscopy of live immobilized cells in culture to whole-animal imaging, are providing insight into *T. brucei* biology, parasite-host interplay, trypanosome CNS invasion and disease progression. We also consider how imaging tools can be used for candidate drug screening purposes that could lead to new chemotherapies.

## Introduction

Human African trypanosomiasis (HAT) is caused by infection with African trypanosomes (*Trypanosoma brucei*), which are transmitted by the haematophagous tsetse fly (*Glossina* spp.). Two subspecies are human infective: *Trypanosoma brucei gambiense*, which is present in West and Central Africa, and *Trypanosoma brucei rhodesiense*, which is found in East and Southern Africa. Human African trypanosomiasis is characterized by two stages, the early or haemolymphatic stage where trypanosomes proliferate at the bite site, travel to local lymph nodes and establish infection in the bloodstream, and the late or meningoencephalitic stage in which trypanosomes cross the blood–brain barrier (BBB) invading the central nervous system (CNS). This late stage invariably leads to coma and death if untreated 1,2. Human African trypanosomiasis has had a severe social and economic impact across sub-Saharan Africa with an estimated 70 million people at risk of infection. Investment in control strategies over the last decade has led to a significant decline in recorded HAT cases with <10 000 cases reported to WHO in 2009 for the first time in 50 years 3. This trend has continued in 2010 and 2011, and attaining the WHO roadmap target for the elimination of African trypanosomiasis (<1 case/10 000 population) is forecast for 2020 4. However, historically, HAT has been characterized by episodic epidemics and resurgences; therefore, control efforts such as surveillance, early diagnosis and treatment of domestic animals to reduce *T. brucei* reservoirs and tsetse fly control must be maintained to prevent another HAT resurgence. In 2009, 76% of reported HAT cases were found in the Democratic Republic of Congo (DRC) 5. In the DRC and other regions experiencing war and civil unrest, accurate reporting of HAT is problematic and this could result in significant underestimation of disease prevalence 6. Research efforts must be continued towards the development of safer, more effective chemotherapy to tackle the disease. Late-stage *T. b. rhodesiense* HAT is currently treated with highly toxic melarsoprol, causing 8·4% of deaths with the new 10-day regimen 7. Late-stage *T. b. gambiense* is now predominantly treated with nifurtimox–eflornithine combination therapy (NECT), a safer option 8 but expensive and difficult to administer 9. In addition, improvement in late-stage diagnostics is necessary as currently an invasive lumbar puncture is required for CSF microscopic analysis and there is no consensus on criteria defining late-stage infections. HAT clinical features, diagnosis and treatment have recently been reviewed 10.

In addition to their clinical relevance, African trypanosomes are also a powerful model organism for research on eukaryotic cell biology, including mechanisms of cell division, intracellular trafficking and organelle biogenesis 11–14. Of particular interest is the highly motile flagellum, which consists of axonemal microtubules (9 + 2 doublet organization) extending from the basal body covered in membrane 15–17. The flagellum is very similar in structure and protein composition to primary cilia in other eukaryotes. This review will focus on how imaging technology has advanced our understanding of African trypanosomes, both as a model system and as an important human pathogen.

## *In vitro* African Trypanosome Imaging

African trypanosome morphology and ultrastructure have been extensively studied using classic light microscopy, transmission electron microscopy (TEM) and scanning electron microscopy (SEM). These studies have revealed that *T. brucei* are curved elongated cells ranging from 12 to 35 μm in length and 1·5 to 3·5 μm in width depending on life cycle stage 18. Trypanosomes and other kinetoplastid species are characterized by the disc-shaped kinetoplast, a network of mitochondrial circular DNA inside a single large mitochondrion. Adjacent to the kinetoplast is the flagellar pocket, an invagination of the plasma membrane which is the major site of exo-and endocytosis due to constraints imposed by the tightly packed subpellicular microtubules 19,20. A single motile flagellum originates at the basal body and emerges from the flagellar pocket extending anteriorly for the entire length of the trypanosome. Movie S1 shows the movement of bloodstream form (BSF) *T. brucei* incubated with human blood. Trypanosome cell architecture is reviewed in Ref. 21, and *T. brucei* imaging protocols have been described in Ref. 22.

During its life cycle, *T. brucei* alternates its environment between its mammalian host and tsetse fly vector. This requires many developmental changes that are precisely programmed and interconnected including alterations in size and shape, surface coat, mitochondrion development and kinetoplast position 21,23,24. Immunofluorescent analyses using cell cycle stage–specific and cytoskeletal markers have given insights into the differentiation process from the procyclic trypomastigote stage in the tsetse fly midgut to the salivary gland epimastigote stage. This requires an asymmetrical cell division cycle, which results in the generation of one long and one short daughter cell with complete remodelling of the subpellicular microtubules prior to division. The short epimastigote is able to proliferate and attach to epithelia on reaching the salivary glands, while the long epimastigote appears to be a dead-end morphological stage 25,26. Of particular interest is the mechanism by which *T. brucei* evades the mammalian host immune response by switching their variable surface glycoprotein (VSG) coat by a process known as antigenic variation, allowing them to maintain a chronic infection 27–30. This clever evasion strategy has severely hindered the development of a protective vaccine against HAT 31.

**Figure 1:**
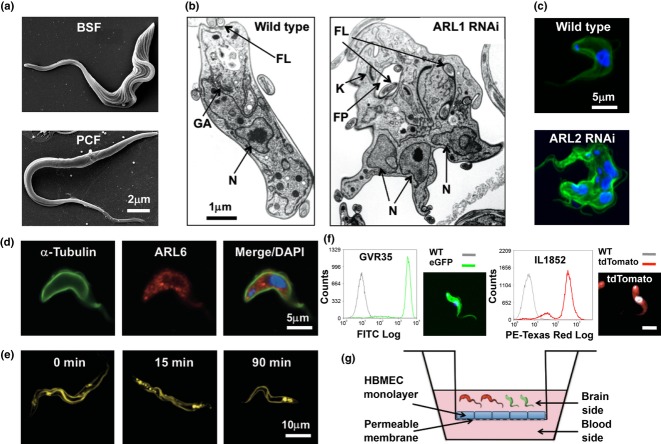
*In vitro* African trypanosome imaging. (a) Scanning electron micrographs of bloodstream form (BSF) *Trypanosoma brucei brucei* strain Lister 427 wild type (top) and procyclic form (PCF) *T. b. brucei* strain EATRO 1125, (bottom). Bar 2 μm. These images were originally published in Ref. 32. (b) Transmission electron micrographs of *T. b. brucei* BSF parasites, wild type (left) (originally published in Ref. 34) and following tetracycline-induced knockdown of *ARF1* for 24 h (right). FL, flagellum; FP, flagellar pocket; N, nucleus; K, kinetoplast; GA, Golgi apparatus. Bar 1 μm. (c) *T. b. brucei* BSF parasites probed with anti-α-tubulin (green) and costained with DAPI. Wild type (top) and following tetracycline-inducible RNAi knockdown of the small GTPase *ARL2* (bottom). Bar 5 μm. (d) Immunofluorescence analysis of *T. b. brucei* BSF to determine subcellular localization of ARL6. These images show a dividing cell (one nucleus and two kinetoplasts) probed with anti-TbARL6 (red) and anti-α-tubulin (green) costained with DAPI (blue). Bar 5 μm. (e) Trafficking of the lipophilic dye FM4-64 in *T. b. brucei* PCF. Cells were washed in PBS and treated with 40 μm FM4-64 (Life Technologies Ltd, Paisley, UK) prior to CyGEL immobilization. Samples were visualized by confocal microscopy, with FM4-64 excited at 543 nm and emission collected through a 560 nm long pass filter. The dye was seen on the plasma membrane and at the flagellar pocket at time 0. By 15 min, the signal appeared in the endosomal system, reaching the terminal lysosome by 90 min. These images were originally published in Ref. 48. (f) Analysis of transgenic *T. brucei* expressing fluorescent proteins. Flow cytometry analysis and visualization by confocal microscopy of *T. b. brucei* GVR35 expressing eGFP and costained with DAPI (left panel). Ninety-nine percentage of cells express high levels of eGFP. Flow cytometry analysis and visualization by confocal microscopy of *T. b. rhodesiense* IL1852 expressing pHD1034 tdTomato costained with DAPI (right panel). 98% of cells express high levels of tdTomato. Transformants were selected with 0·1–1 μg/mL puromycin. Bar 5 μm. (g) Imaging *T. brucei* blood–brain barrier (BBB) transmigration *in vitro*. Transgenic fluorescent parasites crossing the BBB in an *in vitro* transwell model [monolayer of immortalized human brain microvascular endothelial cells (HBMEC)] can be captured in real time by multiphoton microscopy using a collagen gel to plug the basolateral chamber.

The morphological differences between parasite stages can be demonstrated clearly using electron microscopy (EM). Scanning electron micrographs of the mammalian BSF *T. b. brucei* and the more elongated procyclic form (PCF), characteristic of the tsetse fly gut form, are shown in Figure 1 32. Electron microscopy can be used to examine morphological phenotype following molecular manipulations such as RNA interference (RNAi) and gene deletion by homologous recombination. This is illustrated in transmission electron micrographs of *T. b. brucei* BSF cells from Lister 427 wild type 33 (Figure 1b left 34) and of a RNAi mutant line following tetracycline-induced knockdown of *ARF1* (ADP-ribosylation factor 1) for 24 h (Figure 1b right, H. Price unpublished data). Expression of the *ARF1* gene is essential for viability of bloodstream *T. brucei*, and TEM was used to demonstrate that parasites depleted of ARF1 protein exhibit a number of morphological abnormalities prior to cell death, including an enlarged flagellar pocket, multiple nuclei and internal flagella 35. More recently, three-dimensional imaging by electron microscope tomography (three-dimensional reconstructions based on serial section electron micrographs) has provided a wealth of information on key processes and organelle structures such as the mechanisms driving mitosis and cytokinesis (reviewed in Ref. 36), the structure of the flagellum and understanding its bihelical motion 37,38, the kinetoplast duplication cycle 39 and the cellular architecture of the flagellar pocket and basal body 40.

Epifluorescence microscopy and confocal microscopy are now routinely used to image transgenic parasites expressing fluorescent reporter proteins (live, immobilized or fixed) and cells labelled with specific antibodies. These tools are highly informative for phenotype determination following RNAi or gene deletion and for subcellular localization studies. Analysis of DAPI-stained *T. brucei* cells can be used in combination with flow cytometry and SEM to determine at which stage specific proteins play a role in the cell cycle 41,42. Figure 1(c) shows confocal images of wild type and *ARL2* (ADP-ribosylation factor-like 2) RNAi BSF *T. brucei* labelled with DAPI and an anti-α-tubulin antibody (H. Price, unpublished data). Immunofluorescence and SEM data revealed that knockdown of the small GTPase *ARL2* causes a defect in cytokinesis resulting in multinucleated, multiflagellated cells 43. Indirect immunofluorescence and transmission immunoelectron microscopy techniques were employed to find the subcellular localization of another small GTPase ARL6 in BSF *T. b. brucei* (Figure 1d; H. Price, unpublished data) 44. In humans, ARL6 is encoded by *BBS3*, one of 17 genes implicated in the genetic disorder Bardet–Biedl syndrome. ARL6 binds directly to the BBSome protein complex and is required for recruitment of this complex to primary cilia 45. Unexpectedly, imaging studies in *T. brucei* show that ARL6 in the parasite is not associated with the flagellum, but instead is located on electron-dense vesicles throughout the parasite cell body 44.

## Live Cell Imaging

Live cell imaging is an invaluable tool for real-time analysis of fundamental processes, including molecular trafficking. It is now possible to subject live kinetoplastid parasites to advanced microscopy techniques such as fluorescence recovery after photobleaching (FRAP) and fluorescence resonance energy transfer (FRET) for the analysis of molecular diffusion and protein complexing 46. Development of live imaging techniques has allowed the study of highly dynamic processes in the parasite in real time, such as intraflagellar transport (IFT), a flagellum-/cilium-specific mechanism for conveying molecules to and from the distal tip of this organelle within defined particles. FRAP and spinning disc microscopy have recently been used to measure the speed of IFT particle movement in the flagella of *T. brucei* procyclic cells expressing green fluorescent protein (GFP)-tagged IFT52 47. This elegant study demonstrated that IFT movement is extremely fast in trypanosome flagella and showed the presence of at least two independent populations of anterograde IFT trains travelling on single flagella, with common fusion events between the two. Mathematical modelling using these data predicted that only 45% of the IFT protein pool is actively used in flagellar transport 47, indicating that these proteins may have other functions in the cell.

One obvious problem with live imaging of trypanosomes is that the cells have highly motile flagella, and for most types of imaging, they need to be firmly immobilized without compromising viability. Recently, we validated a new rapid method for immobilizing *T. brucei* and *Leishmania* parasites using a thermoreversible gel CyGEL (Biostatus Ltd, Shepshed, UK) 48. CyGEL is liquid when ice-cold but forms a solid matrix upon warming to 15°C and above. *Leishmania major* and PCF *T. b. brucei* maintained a high level of cell viability (>95%) following a 2-h incubation in CyGEL. Unfortunately, immobilization of BSF *T. brucei* in CyGEL caused 90% death within 1 h 48, supporting other studies that have shown that motility is essential for viability in BSF *T. brucei* 13. However, this technique does allow reproducible live cell imaging in *L. major* and PCF *T. brucei,* thereby facilitating real-time capture of fundamental eukaryotic cellular processes at the individual cell level.

We have used live cell imaging and FRAP analyses in the related kinetoplastid *Leishmania* to study the export pathway of hydrophilic acylated surface protein B (HASPB). Hydrophilic acylated surface protein B is only expressed in infective parasite stages 49,50 and, together with small hydrophilic endoplasmic reticulum–associated protein (SHERP), is essential for metacyclogenesis in the sand fly vector 51. Hydrophilic acylated surface protein B is also immunogenic and a promising vaccine candidate 52,53. Hydrophilic acylated surface protein B is a ‘nonclassically’ secreted protein, requiring N-terminal dual acylation for trafficking to the plasma membrane 54. To address the mechanism involved, we used FRAP to investigate lateral diffusion of a reporter fusion protein HASPB18-GFP that is detected on the parasite body, plasma membrane, flagellar pocket and flagellum. Small regions of interest (ROI) within the plasma membrane were photobleached, and the rate of mobility and percentage recovery were used as an indication of the speed of movement and the percentage of mobile vs. immobile molecules. In addition, the corresponding fluorescence loss induced by photobleaching (FLIP) in additional regions outside of the bleached ROI allowed the analysis of HASPB18-GFP movement direction. FRAP of a small ROI at the cell body plasma membrane showed recovery at 6·4 ± 4·8 s, and corresponding FLIPs detected on both sides of the ROI illustrated that HASPB18-GFP can undergo bidirectional movement within the inner leaflet of the membrane [Movie S2; 55]. Interestingly, FRAP over the main cell body (leaving the external section of the flagellum unbleached) revealed rapid recovery of fluorescence (6 s) in a short region of the flagellum within the flagellar pocket, while fluorescence in the plasma membrane did not recover by 37 min following photobleaching. This suggests the presence of a diffusion barrier at the base of the *Leishmania* flagellum that restricts the movement of flagellar proteins to the cell body membrane [Movie S3; 55]. CyGEL immobilization of parasites coupled with FRAP analysis will be a valuable tool for studying this barrier and to gain a better understanding of the mechanism of flagellar trafficking in *T. brucei* and *Leishmania*.

CyGEL can also act as a controlled delivery system for a range of fluorescent probes to PCF *T. brucei* and *L. major*. These include the intercalating agent propidium iodide (excitation/emission maxima: 535/617 nm) to evaluate cell viability and subcellular markers Lysotracker, Mitotracker and FM4-64 [N-(3-triethylammoniumpropyl)-4-(6-(4-(diethylamino) phenyl) hexatrienyl) pyridinium dibromide] 48. We monitored the uptake of the lipophilic styryl dye FM4-64 (excitation/emission maxima: 515/640 nm; Life Technologies, Paisley, UK) by PCF *T. brucei*. Cells were immobilized in CyGEL and incubated with 40 μm FM4-64 over a 90-min time course. *Trypanosoma brucei* were imaged using a Zeiss LSM510 Meta confocal microscope. During this time, the dye moves from cell body membrane and flagellar pocket to the lysosome via the endosomal system (Figure 1e; 48).

Multiphoton microscopy is particularly useful for live cell imaging as the longer wavelength and low-energy excitation lasers are less harmful, allowing cells to be imaged for longer periods than with confocal microscopy. Used typically in 2-photon mode, this approach has a significant advantage in only exciting fluorophores at the focal point of the excitation beam, negating the requirement for a pinhole and improving the efficiency of excitation/emission power 56. These advantages, combined with reduced light scattering and adsorption of excitation light in the near infrared spectrum, improve imaging at depth in complex (e.g. organoid) cultures 56. Combining this advanced imaging technology with the generation of transgenic fluorescent *T. brucei* and an *in vitro* BBB model 57,58 allows us to tackle the challenge of mimicking and investigating CNS invasion by African trypanosomes *in vitro*, the critical event in HAT disease. Little is known about the mechanism of trypanosome CNS invasion. Access to post-mortem human brain tissue is very limited; therefore, the majority of work in this area has been performed with mouse models and *in vitro* studies. The BBB is composed of endothelial cells that line the cerebral microvessels surrounded by basal lamina and astrocytic perivascular endfeet. Brain microvascular endothelial cells (BMEC) form more complex tight junctions than endothelial cells in peripheral tissues, thereby restricting the movement of even small molecules 59. It is thought that both parasite and host factors affect BBB integrity. It has been proposed from studies using an *in vitro* BBB model that brucipain, a trypanosome cysteine protease, induces calcium activation signals in BMEC via protease-activated receptors (PARs), thereby causing brain endothelial barrier dysfunction that allows trypanosome BBB transmigration 58.

It is possible to use stably transfected *T. brucei* 60 as a tool to study *T. brucei* BBB transmigration in real time. We used DNA constructs based on the plasmid vector pHD1034 61,62 (which integrates into the parasite genome downstream of the ribosomal RNA promoter), in order to produce transgenic lines constitutively expressing fluorescent proteins in all parasite life cycle stages. Using the efficient AMAXA nucleofection transfection protocol 63, we have generated *T. b. brucei* GVR35 (strain used in the murine late-stage HAT model 64) expressing eGFP (excitation/emission maxima: 488/507 nm) and *T. b. rhodesiense* IL1852 (originally isolated from the CSF of a patient in Kenya) expressing pHD1034 tdTomato (excitation/emission maxima: 554/581 nm) (Figure 1f; L. MacLean, unpublished data; plasmid pHD1034, a gift from Z. Mackey, Virginia Tech, USA). Ongoing work involves incubation of these fluorescent parasites in the blood side of the BBB model transwell (composed of a monolayer of immortalized human BMEC, a gift from K. S. Kim, D. Grab, Johns Hopkins University, USA), the addition of collagen gel to plug the basolateral chamber and imaging parasite transmigration in real time using multiphoton microscopy (Figure 1g). Generating such *in vitro* model systems is important in order to gain insights into this poorly understood, yet critical event in HAT leading to morbidity and mortality.

*In situ* imaging of transgenic *T. brucei* lines expressing fluorescent proteins has become a valuable tool for studying the development of the parasite in the tsetse fly vector. Studies on flies co-infected with two parasite lines expressing either GFP or red fluorescent protein showed that a subpopulation of yellow hybrids are produced in the salivary glands of the vector. This provided the first evidence showing the site at which *T. brucei* genetic exchange occurs, knowledge of direct relevance to our understanding of the population genetics of the parasite 65,66.

## *In vivo* African Trypanosome Imaging

Although *in vitro* models are invaluable tools, *in vivo* studies are required to provide insight into host–parasite interplay and to reveal details of the mechanisms involved in *T. brucei* CNS invasion and subsequent disease progression. Evidence from HAT mouse models reveals that the host neuroinflammatory response plays a role in determining trypanosome entry into the CNS. In *T. b. brucei*-infected mice, IFN-γ was shown to facilitate T-cell and parasite crossing of the *glia limitans* (basement membrane), and parasites were captured between the two sets of basement membranes in IFN-γ or IFN-γ receptor–deficient mice 67. Furthermore, our work on HAT pathogenesis in Uganda found that high levels of systemic IFN-γ in patients were associated with increased disease severity 68. A recent study has employed contrast-enhanced magnetic resonance imaging (MRI) to show that the integrity of the BBB is compromised during late-stage trypanosomiasis in the mouse model and this is not associated with the presence of a severe neuroinflammatory reaction 69. Until this point, the effects of trypanosome infection on BBB integrity remained equivocal with one study, in a late-stage rat model of infection, detecting neither loss of the tight junction proteins occludin or zona occludens-1 nor leakage of albumin into the brain 70. In contrast, a second investigation found a significant and progressive BBB leakage demonstrated by the presence of a fluorescent dye in the brain parenchyma following peripheral intravenous injection 71. MRI has several benefits over these more conventional histological or biochemical techniques. For example, MRI allows imaging of the whole brain and can identify specific regions of altered BBB integrity. Furthermore, as the process is conducted *in vivo*, this precludes the occurrence of artefacts that could result following the death of the animal. However, perhaps the greatest advantage of MRI is the ability of the technique to monitor the integrity of the barrier in a single animal over a predefined time course of infection or treatment. This methodology has been successfully applied to demonstrate the rapid restoration of BBB function following a curative course of a novel oral formulation of the trypanocidal drug melarsoprol 72. Because MRI has shown a significant reduction in BBB integrity following trypanosome infection, it would be logical to assume that early-stage drugs could gain entry to the CNS and effectively cure late-stage disease. Unfortunately, this is not the case, as although treatment with early-stage drugs does reduce parasite burden within the brain (J. Rodgers, unpublished data), a population of parasites remains following treatment. By combining MRI with the state-of-the-art microscopy techniques detailed in this review, it may be possible to identify the areas within the CNS that harbour this residual population and select these areas as specific ROI in MRI analyses. Furthermore, by tracking parasite transmigration into the CNS, it may be feasible to determine whether the BBB leakage associated with trypanosome infections is a direct product of trypanosome traversal of the barrier or a product of the ongoing peripheral inflammatory response.

Thus far, there is a paucity of data on the route of *T. brucei* primary CNS invasion and the sequential movement and maintenance of parasites within the brain as disease progresses. A previous comprehensive morphological study of experimental cerebral trypanosomiasis 73 analysed the histopathology of mouse brain serial sections from animals infected with *T. b. brucei* LUMP 227 over 1–9 weeks using haematoxylin and eosin staining. Immunofluorescence was also performed on frozen brain sections, where parasites were labelled with antitrypanosomal antibodies (against LUMP227 antigens) and detected by a fluorescein-conjugated secondary antibody. The histopathology revealed that parasites are first found in the choroid plexuses (lateral and fourth) in weeks 3–4, mainly in the interstitium in the lumen and wall of the vessels, and a small number are found in the meninges usually at week 6. By immunofluorescence, parasites were detected slightly earlier in the choroid plexuses from week 2 and in the meninges from week 4. In advanced cases, large numbers of parasites were found in the choroid plexuses, in perivascular spaces and occasionally in the white matter. Poltera *et al*. therefore proposed that the route of parasite invasion of the brain may be by migration from the vascular compartment to the choroid plexus interstitium, aided by the increased permeability of choroid plexus vessels compared to cerebral vessels 74, with subsequent invasion of additional brain structures following the initiation of a local inflammatory reaction within the choroid plexus.

**Figure 2:**
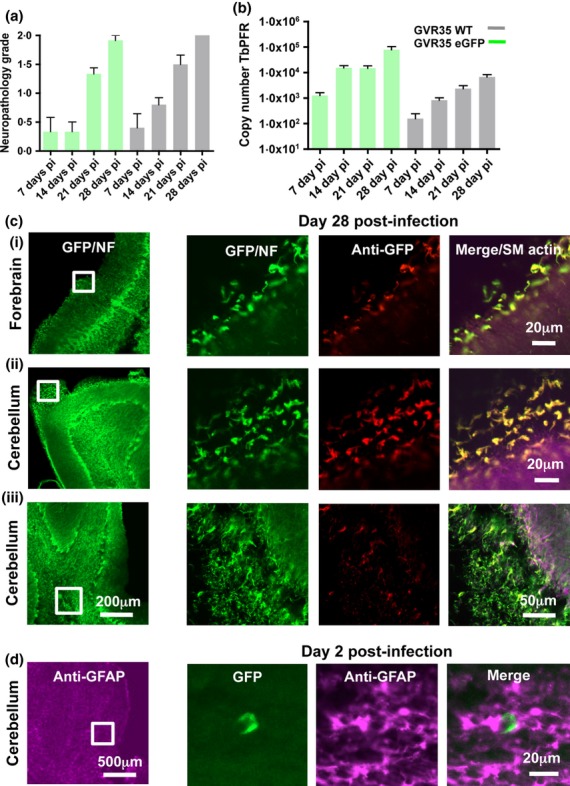
Detection of *Trypanosoma brucei brucei* GVR35 eGFP in the central nervous system (CNS). *T. b. brucei* expressing GVR35 eGFP were passaged through a female CD-1 mouse by intraperitoneal inoculation of 1 × 10^5^ parasites in HMI-9 media. On day 10 post-infection, the trypanosomes were harvested and 4 × 10^4^ parasites passaged through a second mouse. After 28 days, the mouse was exsanguinated by cardiac puncture and the brain was excised [All *in vivo* experiments were authorized in the United Kingdom under the Animals (Scientific Procedures) Act 1986 and approved by the University of Glasgow Ethical Review Committee]. (a) Graphical representation of neuropathology grade over days 7–28 post-infection with *T. b. brucei* GVR35 wild type (grey) and eGFP transgenic parasites (green). The severity of neurological reaction to *T. brucei* infection was determined by a neuropathology grading scale from 0 to 4. Briefly, haematoxylin and eosin–stained sections through the hippocampal brain region were examined. Normal brain was classified as grade 0, mild meningitis as grade 1, moderate meningitis with some perivascular cuffing as grade 2, more severe meningitis and perivascular cuffing with some inflammatory cells in the neuropil as grade 3 and severe meningoencephalitis with inflammatory cells throughout the parenchyma as grade 4. (Boxes represent mean values, and whiskers represent standard error.). (b) Graphical representation of parasite load in mouse brain over days 7–28 post-infection with *T. b. brucei* GVR35 wild type (grey) and eGFP transgenic parasites (green). Parasite load was determined by Taqman-PCR to detect the *T. brucei* paraflagellar rod (*PFR*) *2* gene using whole brains. (c) Confocal microscopic analysis of whole-mount day 28 post-infection brain sections. Following fixation in 0·4% paraformaldehyde, the whole brain was embedded in 8% agarose in PBS and axial sections (200 μm thick) were obtained using a Leica VT 1000S vibratome. Tissue sections were blocked in 5% goat serum/0·15% Triton X-100/PBS for 60 min and then incubated with primary antibodies, mouse antismooth muscle actin (Sigma-Aldrich Co Ltd) to detect the vasculature and rabbit antineurofilament 200 (Sigma-Aldrich) at a dilution of 1 : 500, for 24 h. Sections were washed with 0·15% Triton PBS × 3 and left overnight in wash buffer at 4°C. Secondary antibodies goat anti-mouse Alexa 594 (pink) and goat anti-rabbit Alexa 488 (green) were added at a dilution of 1 : 500 and incubated for 24 h. Sections were washed as above and blocked in 5% rabbit serum/0·15% Triton X-100/PBS for 60 min, then incubated with rabbit anti-green fluorescent protein (GFP) Alexa 647 (red) at 1 : 500 dilution (Life Technologies) for 24 h. Sections were again washed as above, fixed in 4% PFA for 15 min at room temperature and washed three times over 30 min. Sections were gradually dehydrated in successive concentrations of methanol in PBS–Triton X-100 and subsequently incubated in benzyl alcohol/benzyl benzoate (Sigma-Aldrich Co Ltd) (BABB) with MeOH to a dilution of 50% BABB and 50% MeOH for 10–15 min at room temperature in glass containers. Finally, the sections were left in 100% BABB for 25 min and mounted in metal slides made with a large opening drilled through the centre to which a coverslip was attached to one side using nail varnish. The depression was filled with fresh BABB and the sample transferred to the slide. Another coverslip was placed on top and sealed with nail varnish. Sections were analysed using a Zeiss LSM710 Meta invert confocal microscope using a EC Plan-Neofluar 10×/0·3 and LD C-Apochromat 40×/1·1W objective lens. The white box in the left-hand image represents the region shown at higher magnification in the four images on the right. *T. b. brucei* GVR35 expressing eGFP were shown to be present in the meninges in the forebrain (ci) and cerebellum (cii) and within the brain parenchyma (ciii) at day 28 pi. This was confirmed by rabbit anti-GFP Alexa 647 labelling (red). (d) Confocal microscopic analysis of whole-mount day 2 post-infection brain sections. Axial thick brain sections were prepared as in Figure 2c; however, sections were labelled with rabbit anti-GFAP antibody alone, detected by Alexa 594 (pink) to visualize astrocytes. *T. b. brucei* GVR35 expressing eGFP were detected in the cerebellar parenchyma by day 2 post-infection.

It is now possible to use transgenic fluorescent *T. brucei* in combination with whole-mount labelling of thick tissue sections (100–200 μm) cut using a vibrating blade (vibratome) and confocal microscopy as a sensitive method for detecting *T. brucei* in the brain. GVR35 eGFP clones were first assessed by flow cytometry to quantify the level of fluorescence. In the best clone, 99% of the GVR35 eGFP population were shown to express a high level of eGFP (Figure 1f), and after establishing infection for 28 days, GVR35 eGFP parasites were detected in a wet blood film using fluorescence microscopy (J. Rodgers, unpublished data). The severity of the neurological response to *T*. *brucei* infection was determined by a neuropathology grading scale from 0 to 4 as previously described 75. GVR35 wild type and eGFP transgenic parasites showed comparable levels of neuropathology at days 7–28 post-infection, both exhibiting an increase over time and displaying moderate meningitis with some perivascular cuffing by day 28 (Figure 2a; J. Rodgers, unpublished data). Therefore, the transgenic line is a valid tool for analysing CNS infection. Parasite load was determined in whole brains by real-time quantitative PCR as described previously 72 by amplification of the paraflagellar rod (*PFR*) 2 gene, which is specific to kinetoplastids. Similar trends of increasing parasite load over time were found following infection with wild type and eGFP-expressing GVR35 parasite lines (Figure 2b; J. Rodgers, unpublished data).

Using brain antineurofilament antibody labelling, detected by goat anti-rabbit Alexa 488 (green), it was possible to obtain an overview of day 28 post-infection axial brain sections using confocal microscopy (Figure 2c left panel, white box represents enlarged area in right panel; L. MacLean, unpublished data). Looking at a higher magnification, as detected by eGFP (excitation also: 488 nm, green) and confirmed by anti-GFP/Alexa 647 (red) labelling, it was apparent that eGFP-expressing *T. brucei* were abundant particularly in the meninges of the forebrain (Figure 2c i) and cerebellum (Figure 2c ii) and within the cerebellar parenchyma (Figure 2c iii). Preliminary analysis of brains isolated from mice at day 2 post-infection, labelled with rabbit anti-GFAP 594 (pink) to detect glial fibrillary acidic protein, the main intermediate filament protein expressed by astrocytes, revealed GVR35 eGFP parasites in the cerebellar parenchyma (Figure 2d), suggesting that trypanosome CNS invasion occurs before neurological signs are detected. Studies are underway to analyse early invasion in more detail and to determine parasite distribution kinetics during disease progression using GVR35 eGFP-infected mice. Following labelling, thin sections of brain are being imaged using a fully automated slide scanner, which allows rapid analysis of serial brain sections at several time points. Detection of parasites in the brain very quickly following infection supports Frevert *et al.'s* work using intravital confocal microscopy. In this study, green fluorescent cell tracker dye PKH67-labelled *T. brucei* (excitation/emission maxima: 490/502 nm; Sigma-Aldrich Co Ltd, Dorset, UK) and monomeric mOrange-expressing *T. brucei* (excitation/emission maxima: 548/562 nm; 61,76) were detected in the mouse brain parenchyma within 24 h of infection before significant microvascular inflammation was observed 77. These findings are of particular interest as we have recently reported neurological symptoms in patients presenting with early-stage *T. b. rhodesiense* infections 78.

These studies demonstrate that fluorescent parasites such as GVR35 eGFP will be an invaluable tool for further investigation of African trypanosomes in their mammalian host, including live *in vivo* imaging of late-stage HAT, and, once passaged, in the tsetse fly transmission vector.

## Whole-Animal *In Vivo* (IVIS) Imaging

The development of live whole-animal imaging and transgenic mouse models has been particularly useful in visualizing *T. brucei* dissemination in the host from the inoculation site to local lymph nodes, establishing bloodstream infection and subsequently CNS infection. Imaging studies that allow us to examine host responses to infection at the whole-animal level are of great importance. Various reporter trypanosome lines expressing bioluminescent proteins have been generated over the last few years to enable the noninvasive imaging of trypanosome infections in mice. Claes *et al*. 79 and Giroud *et al*. 80 described the generation of *T. b. brucei* 427, *T. b. brucei* AnTat1.1 and *T. b. gambiense* lines expressing Renilla luciferase. With the use of bioluminescence imaging, the progression and dissemination of infection could be tracked *in vivo* and localization to specific organs shown by *ex vivo* imaging. More recently, the firefly luciferase variant LUC2 (Promega) has also been employed for imaging *T. b. brucei* infections.

**Figure 3:**
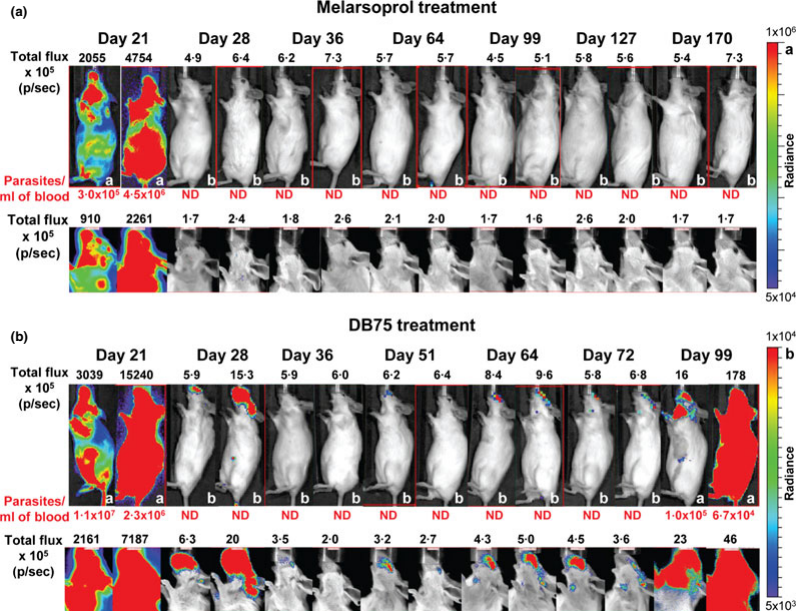
Bioluminescence imaging to assess chemotherapy for stage 2 trypanosomiasis. Mice were injected intraperitoneally with 3 × 10^4^ *Trypanosoma brucei brucei* GVR35-LUC2 bloodstream form (BSF) parasites and treated from 21 days post-infection with (a) melarsoprol (topical application of 3·6 mg on three consecutive days) and (b) DB75 (20 mg/kg on five consecutive days intraperitoneally). Mice were imaged at indicated times after infection using an IVIS Spectrum (PerkinElmer, Waltham, MA, USA). D-Luciferin (150 mg/kg) was injected intraperitoneally 10 min prior to imaging. Bioluminescence is shown as total flux in photons per second (p/s) and the parasitaemia of each mouse is shown below the image with ND indicating that trypanosomes were not detected in blood samples. The same two representative mice are shown over the entire period. Two colour scales are used for both treatments to indicate strong (a) and weaker (b) bioluminescent radiance in photons/s/cm^2^/steradian.

The monitoring of acute infections provides a fast method to test compounds against first-stage trypanosomiasis, but new drugs against the second or CNS stage of the disease are most urgently required. The current model for trypanosome CNS infections makes use of the GVR35 strain of *T. b. brucei*, which establishes CNS infection by 21 days post-infection 64. In the original model, mice are treated with test compounds from 21 days post-infection and monitored for 180 days after treatment by assessing blood parasitaemia. We have modified *T. b. brucei* GVR35 to stably express firefly luciferase (LUC2), thereby allowing the monitoring of CNS-stage infections and chemotherapy by *in vivo* imaging (Figure 3). The substrate for firefly luciferase, D-luciferin, readily crosses the BBB, making this reporter the best choice for high-sensitivity bioluminescence imaging in the brain. Treatment of GVR35-LUC2-infected mice with a curative dose of melarsoprol 81 abolished all bioluminescence. In contrast, in mice treated with DB75 [2,5-bis(4-amidinophenyl)furan], a compound shown to be ineffective against CNS trypanosomes 82, bioluminescence was detected in the heads of treated mice where it increased and spread to cervical lymph nodes and eventually the rest of the body. With the new imaging model, bioluminescent trypanosomes were detected several weeks before detection in the blood, providing a significant improvement on the post-treatment time required to establish whether a promising compound is effective for stage 2 (CNS) trypanosomiasis (E. Myburgh *et al*., manuscript submitted).

## Conclusions

By exploiting recent advances in imaging technology, coupled with developments in molecular and cellular biology, rapid progress has been made in our understanding of the biology of these lethal pathogens. Emerging super-resolution microscopy techniques including stimulated emission depletion (STED), photoactivated light microscopy (PALM) and stochastic optical reconstruction microscopy (STORM) will enable detailed structure to be studied at a higher resolution using light microscopy (LM), thus narrowing the gap between LM and EM. Multiphoton imaging can also be enhanced by using the latest fluorescent protein derivatives that are red-shifted and hence can be imaged at greater depths. These can be excited at longer, deeper penetrating wavelengths and emitted light is able to escape back to the detector from greater depths. In addition, a new technique Clear Lipid-exchanged Acrylamide-hybridized Rigid Imaging/Immunostaining/*In situ* hybridization-compatible Tissue-hYdrogel (CLARITY) has recently been used for imaging and molecular phenotyping of intact tissue 83. Employing these new imaging tools will give critical new insight into the cellular and molecular basis of late (CNS)-stage HAT and may advance future drug development and screening programmes.

## SUPPORTING INFORMATION

Movie S1 Bloodstream form (BSF) *T. b. brucei* in human blood.

Movie S2 Plasma membrane FRAP analysis of *Leishmania* HASPB18-GFP.

Movie S3 Whole-cell-body FRAP analysis of *Leishmania* HASPB18-GFP.
